# In Vitro and In Vivo Anticancer Activity of the Most Cytotoxic Fraction of Pistachio Hull Extract in Breast Cancer

**DOI:** 10.3390/molecules25081776

**Published:** 2020-04-13

**Authors:** Maryam Seifaddinipour, Reyhaneh Farghadani, Farideh Namvar, Jamaludin Bin Mohamad, Nur Airina Muhamad

**Affiliations:** 1 Institute of Biological Sciences, Faculty of Science, University of Malaya, Kuala Lumpur 50603, Malaysia; m.seyfadini@gmail.com; 2 Department of Molecular Medicine, Faculty of Medicine, University of Malaya, Kuala Lumpur 50603, Malaysia; r_farghadani@yahoo.com; 3 Faculty of Medicine, Mashhad Branch, Islamic Azad University, Mashhad 917568, Iran

**Keywords:** pistacia (*Pistacia vera*) hulls, breast cancer, anticancer

## Abstract

Pistacia (*Pistacia vera*) hulls (PV) is a health product that has been determined to contain bioactive phytochemicals which have fundamental importance for biomedical use. In this study, PV ethyl acetate extraction (PV-EA) fractions were evaluated with the use of an MTT assay to find the most cytotoxic fraction, which was found to be F13b1/PV-EA. After that, HPTLC was used for identify the most active compounds. The antioxidant activity was analyzed with DPPH and ABTS tests. Apoptosis induction in MCF-7 cells by F13b1/PV-EA was validated via flow cytometry analysis and a distinctive nuclear staining method. The representation of genes like *Caspase 3*, *Caspase 8*, *Bax*, *Bcl-2*, *CAT* and *SOD* was assessed via a reverse transcription (RT_PCR) method. Inhabitation of Tubo breast cancer cell development was examined in the BALB-neuT mouse with histopathology observations. The most abundant active components available in our extract were gallic acid and the flavonoid quercetin. The F13b1/PV-EA has antiradical activity evidence by its inhibition of ABTS and DPPH free radicals. F13b1/PV-EA displayed against MCF-7 a suppressive effect with an IC_50_ value of 15.2 ± 1.35 µg/mL. Also, the expression of *CAT*, *SOD*, *Caspase 3*, *Caspase 8* and *Bax* increased and the expression of *Bcl-2* decreased. F13b1/PV-EA dose-dependently inhibited tumor development in cancer-induced mice. Thus, this finding introduces F13b1/PV-EA as an effectual apoptosis and antitumor active agent against breast cancer.

## 1. Introduction

The pistachio hull refers to the epicarp which has a reddish/yellow color during development and when it ripens, it is a rosy and light yellow [1,2]. Usually, collected pistachio nuts are encased in this shell which is removed by a dehulling process. During the pistachio dehulling process many types of by-products are generated that are currently considered as an agriculture waste and to a lesser extent, are used as fodder by local livestock farmers. Hulls may also be used as an herbal medicine for stomach pains and the prevention of diarrhea and to improve hemorrhoids. Pistachio hull has caught the attention of researchers in recent years due to its natural phenolic and antioxidant compounds. Recent literature has proven that pistachio hull extracts have antioxidant, antimicrobial and antimutagenicity activities. Several reports have validated and established the pharmacological activities and medicinal properties of pistachio hull [1,3,4]. In a report by Tomaino the antioxidant activity of the polyphenols extracts from natural shelled pistachios (NP) was determined. In the rats treated with NP a remarkable decrease was observed for CAR-induced histological paw damage, nitrotyrosine formation and neutrophil infiltration. These results demonstrated that the polyphenols display antioxidant properties in lower doses [1].

In Goli [4] report pistachio hulls were extracted with three different solvents (water, methanol and ethyl acetate) and its total phenol content were determined using the Folin–Ciocalteu method. Additionally, the effect of water and methanolic extracts on the stability of soybean oil that was heated to 60 °C was ascertained. The pistachio hull extract (PHE) slowed down the process of oil deterioration at 60 °C with a concentration-dependent increase between 0.02–0.06%. The 0.06% PHE showed a similar activity pattern to butylhydroxyanisole (BHA) and butylated hydroxytoluene (BHT) of. Thus, pistachio hulls, which at the moment are mainly considered as agricultural waste, contain antioxidants that may be compatible for adding them to food products [4].

It should be noted that the content of antioxidant compounds could vary depending on the extraction procedures adopted. Indeed, for pistachio it has been demonstrated by Garavand et al. [5], who studied and measured the phytochemical substances and radical scavenging activity of pistachio hull extracts, obtained using diverse solvents (water, ethanol, and butanol). Their results showed that ultrasound-assisted aqueous extraction of the hull using ultrasound power (35 kHz) was more effective in increasing the phytochemicals content than a sonochemical ultrasonication method (130 kHz). The amount of vanillic acid, *p*-coumaric acid, naringenin, and catechin in the ultrasound-assisted extracts increased as demonstrated by high-performance liquid chromatography-mass spectrometry. The content of phenolics and antioxidant properties of the aqueous extract decreased remarkably after post-extraction sonication. Contrariwise, the amount of phenolics and flavonoids improved with microwave-assisted extraction in a power-dependent trend [5]. Grace et al. [6] described the presence of anacardic acids, fatty acids, carotenoids, tocopherols and phytosterols as the main components in pistachio hulls. Quercetin-3-*O*-glucoside together with smaller concentrations of quercetin, myricetin and luteolin flavonoids were found in a polar (P) extract. Gallotannins and other phenolic compounds esterified with a gallic acid moiety were characterized in the P extract. Release of nitric oxide (NO) and reactive oxygen species (ROS) were inhibited by the P extract in lipopolysaccharide-stimulated RAW 264.7 macrophage cells. In addition, in the macrophages the non-mitochondrial oxidative burst associated with inflammatory response were reduced by the P extract [6].

Bulló, et al. [7] also investigated the anti-inflammatory properties of polyphenol extracts from natural raw shelled pistachios (NP). For the determination of the amount of protection offered by NP against lipopolysaccharide (LPS)-induced inflammation, the monocyte/macrophage cell line J774 was utilized. The in vitro study illustrated that pre-treatment with NP decreased the TNF-α and IL-1β production and degradation of IκB-α, although not significantly. These results show that, at lower doses, the polyphenols present in pistachios possess anti-inflammatory properties [7].

The hulls of pistachio have been shown to have in vitro antioxidant and in vivo photoprotective effects [2], and also exhibit antimicrobial and antimutagenicity [8] as well as enzyme inhibitory and also possess radical scavenging activities [4].

Some phytochemical assessments have revealed the presence of wide ranging levels of phenolic and flavonoids compounds such as gallic acid, catechin, cyanidin-3-*O*-galactoside, eriodictyol-7-*O*-glucoside and epicatechin in the skin of pistachio, which is even 10 times richer than the seeds [9].

In our previous study, we have demonstrated a promising cytotoxic effect and anti-angiogenesis potential of the ethyl acetate extract from pistachio (*Pistacia vera*) hulls (PV-EA) against MCF-7 breast cancer cells [10]. Therefore, in the current study, we have taken this research a step further and investigated the anticancer activity of the most cytotoxic fraction of PV-EA through the utilization of in vitro and in vivo models of breast cancer.

## 2. Results

### 2.1. Separation of the Bioactive Compound

**Figure 1:**
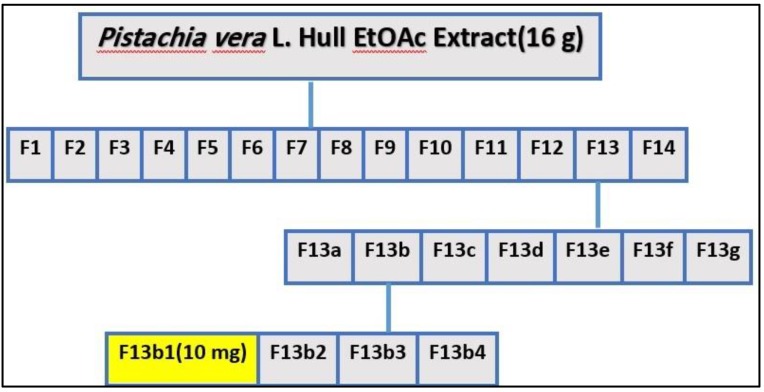
Flow chart from the three steps of bioassay guided fractionation of F13b/PVLH-EAE.

**Figure 2:**
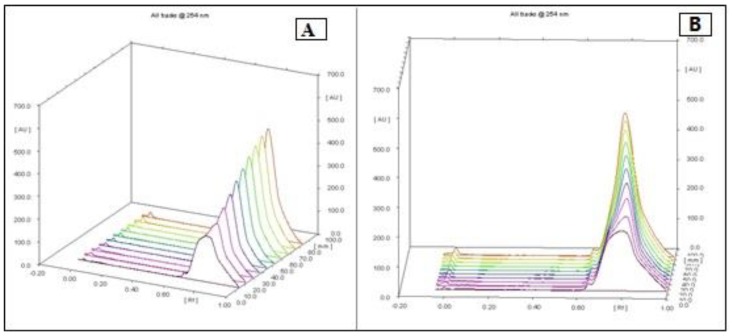
The image and spectrum of all 10 HPTLC spots scanned at 254 nm wavelength on 2 sides (**A**) and (**B**).

**Figure 3:**
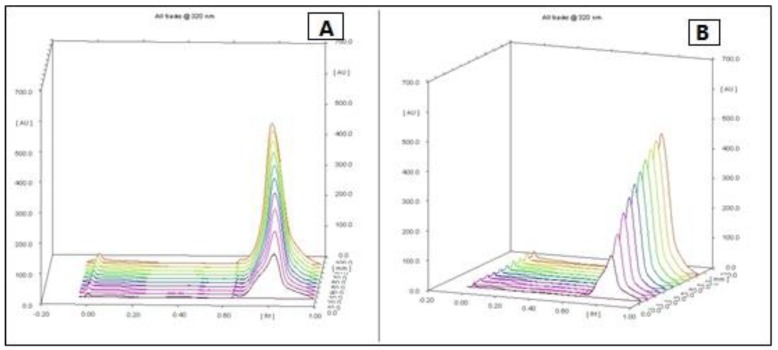
The image and spectrum of all 10 HPTLC spots scanned at 320 nm wavelength on 2 sides (**A**) and (**B**).

**Figure 4:**
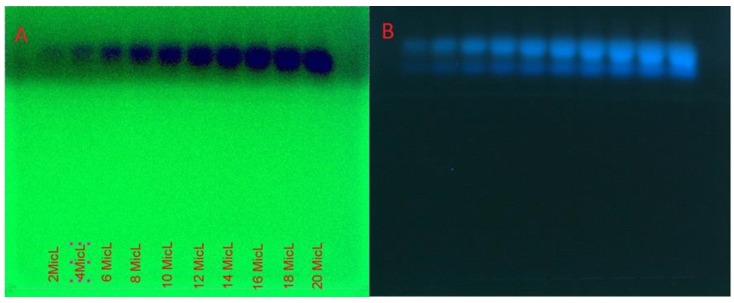
The image and spectrum of all 10 spots scanned at (**A**) 254 nm and (**B**) 320 nm wavelengths.

**Figure 5:**
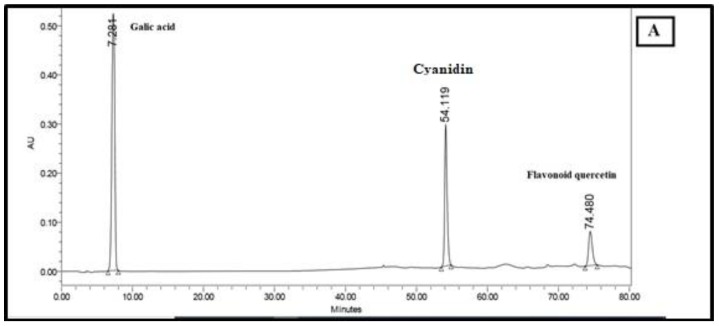

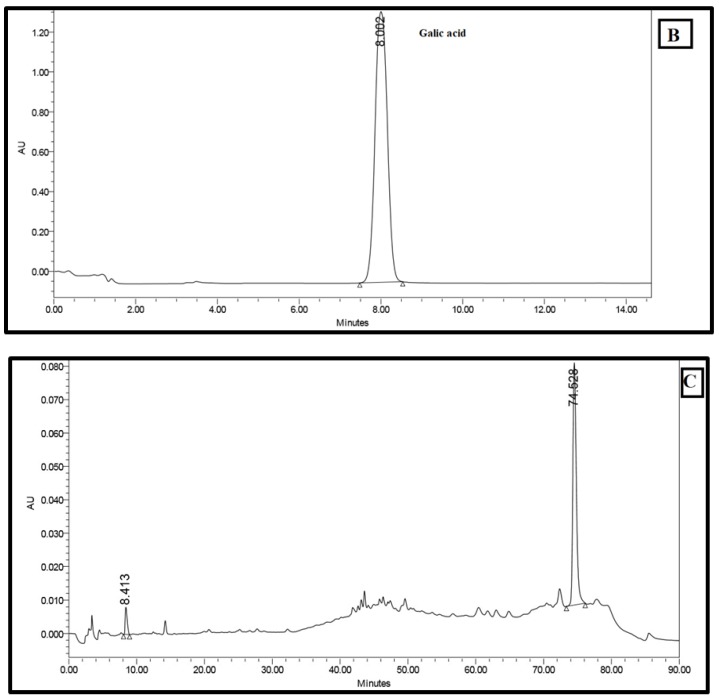
HPTLC analysis. (**A**) Injection of blended standard and, (**B**) Injection of gallic acid standard, (**C**) injection of F13b1/PV-EA to the column.

Dried hulls of *Pistacia vera* were extracted with ethyl acetate. The ethyl acetate extract (16 g) was fractionated in three steps by column chromatography on silica gel 60, which yielded some fractions in each step. After doing an MTT assay and choosing the most cytotoxic fraction in every step, we continued with the next step until the isolation and purification of final fraction (F13b1/PV-EA) that was about 10 mg with an IC_50_ 15.2 µg/mL (Figure 1). Preparative HPTLC using 100% methanol as the mobile phase and silica gel as the stationary phase with 10 concentrations or tracks was done. The image and spectrum of spots were scanned at two wavelengths (254 and 320 nm). All spectra were the same and in an identical region (Figure 2, Figure 3 and Figure 4). Chemical profiling of F13b1/PV-EA was investigated by the use of HPTLC again. After comparing retention times, first with blended standards (gallic acid, cyanidin and the flavonoid quercetin and a second time only with gallic acid and the purified compound from pistachio it was found that gallic acid and quercetin were present in the F13b1/PV-EA fraction (Figure 5).

### 2.2. Cytotoxic effect of F13b1/PV-EA toward MCF-7 Cells

An evaluation of the cytotoxic properties of F13b1/PV-EA in the MCF-7 cell line was performed using the prescribed MTT assay.

**Figure 6:**
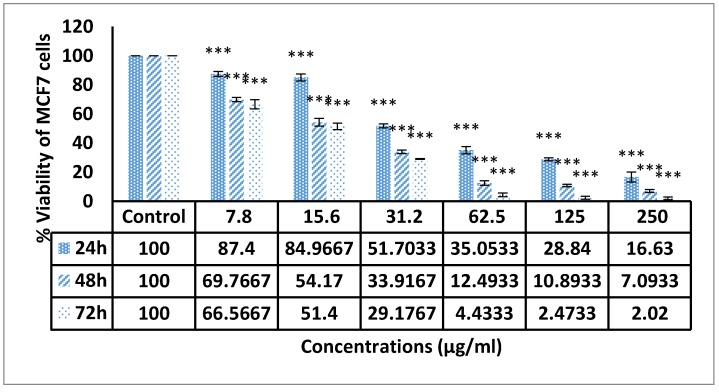
Shows the growth inhibition effects of F13b1/PV-EA on MCF-7 cells noted at different intervals (24, 48 and 72 h) and concentrations. (*** *p* value < 0.001). All of the in vitro experiments were done in triplicate.

Different concentrations ranging from 7.8 to 250 μg/mL of the compound were used and the amount of formazan formed was specified and detected after 24, 48 and 72 h of incubation. Figure 6 display that F13b1/PV-EA resulted in dose-dependent and time-dependent decline in cell viability with increasing concentration and treatment period. The results suggest that cell growth was prevented when the cells were incubated in the presence of the compound.

### 2.3. Apoptotic Morphological Variations

**Figure 7:**
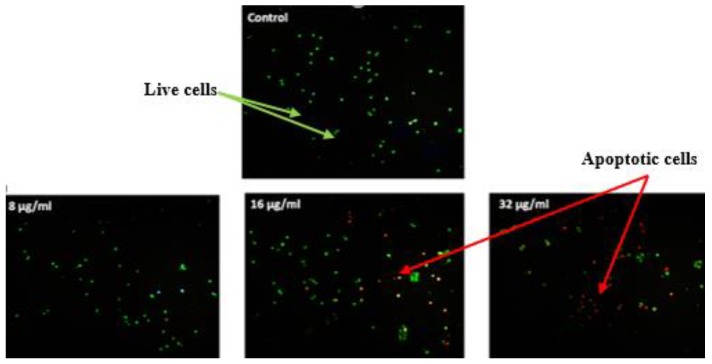
Fluorescent images of MCF-7 cells dyed by AO/PI. Untreated cells (×200) and treated with three concentration of F13b1/PV-EA for 48 h (×200).

**Figure 8:**
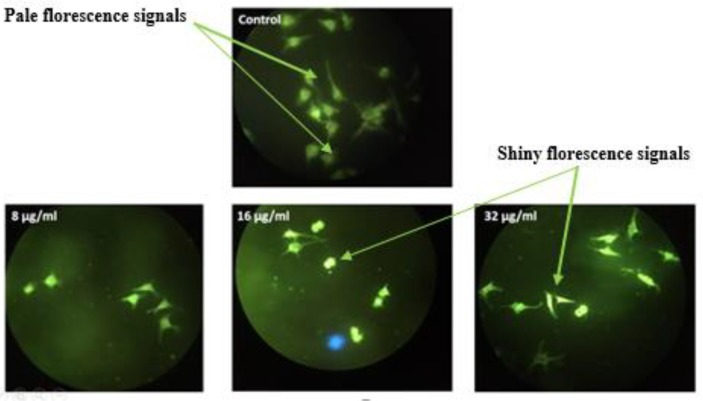
Fluorescent images of Hoechst 33342 stained MCF-7 cells. Untreated cells (×200) and cells treated with 8, 16 and 32 µg/mL of F13b1/PV-EA for 48 h (×200).

Figure 7 shows the results acquired after performing the AO/PI tests. From the data, it can be seen that the compound has dose-dependent effects on cell viability and induces apoptotic morphological variations in treated cells. The results show reduced viability as more apoptotic cells (red in color) were seen at all three concentrations of treatment. In addition, Hoechst 33342 staining (Figure 8), also revealed that the F13b1/PV-EA stimulates apoptotic morphological variations. The cells underwent amazing nuclear changes when treated. However, in the untreated group, the cells were uniformly stained by the fluorescence Hoechst dye indicating the nuclei of the cells were virgin. However, with increasing concentration level of the compound, there was an increase of intensity captured on fluorescence signals and luminous points where the cells expressed apoptotic morphological variations.

### 2.4. Flow Cytometer Analysis

**Figure 9:**
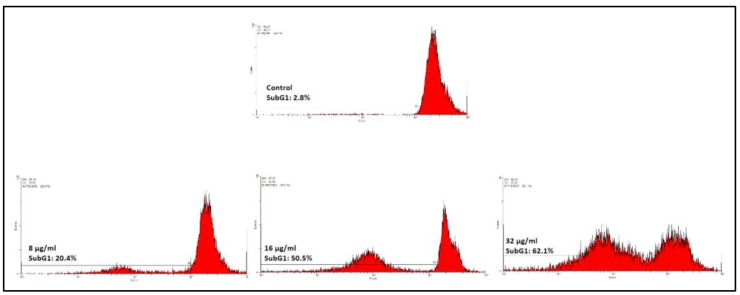
MCF-7 cell cycle analysis of untreated cells (×200) and cells treated with 8, 16 and 32 µg/mL of F13b1/PV-EA for 48-h interval (×200).

By utilizing PI staining, we tried to establish whether MCF-7 cells treated with F13b1/PV-EA underwent apoptosis accompanied by alteration in the cell cycle, and the distribution index was also noted. This was in tandem with growth in the Sub-G1 population with increasing concentrations as shown in Figure 9. As depicted in the mentioned figure, high concentration treatment with the compound (32 µg/mL) led to a growth in the percentage of Sub-G1 phase up to 62.1% ± 0.41 when compared to the control cells which were at 2.8% ± 0.86%, thus indicating a change in arrested cells towards a Sub-G1 population which is known as apoptotic cells. The population of cells that possesses sub-diploid DNA content is a clear indication of DNA fragmentation happening at the time of apoptosis.

### 2.5. RT-PCR Evaluation

The link of some genes containing *Caspase 3*, *Caspase 8*, *Bax*, *Bcl-2*, *CAT* and *SOD* with apoptosis induced by F13b1/PV-EA were observed using RT-PCR.

**Figure 10:**
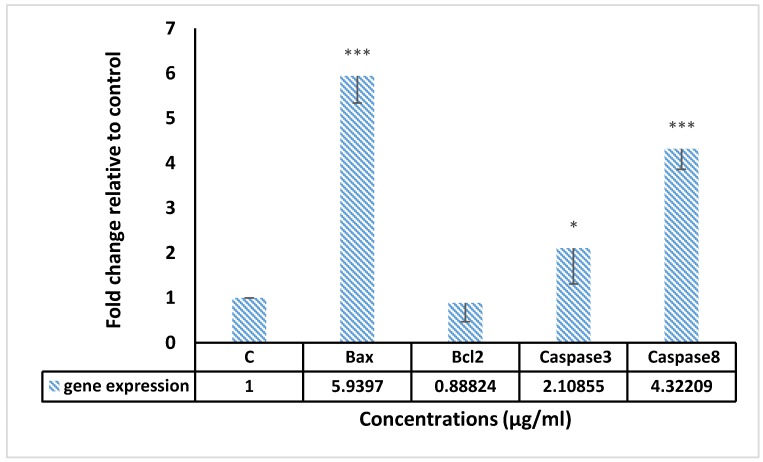
The *Bax*, *Bcl-2*, *Caspase 3* and *Caspase 8* genes expression of MCF-7 cells treated with 15 µg/mL of F13b1/PV-EA for 24 h. (* *p* < 0.05, *** *p* < 0.001).

**Figure 11:**
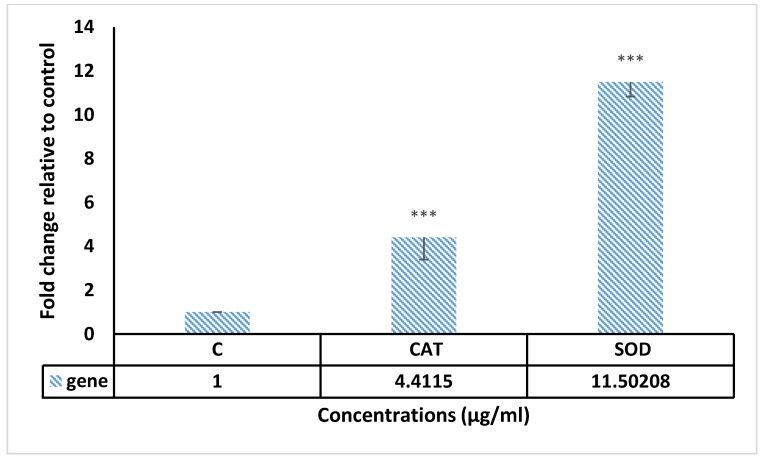
The *CAT* and *SOD* genes expression of MCF-7 cells treated with 15 µg/mL of F13b1/PV-EA for 24 h. (*** *p* value < 0.001).

As explained in Figure 10 and Figure 11, *Caspase 3*, *Caspase 8*, *CAT*, *Bax* and *SOD* gene expressions increased, respectively, when compared to the control (gene expression in cancer cells without any treatment). Further investigation revealed that the compound treatment lowered the expression level of *Bcl-2* over time. These results show that F13b1/PV-EA could stimulate apoptosis by shifting the regulation of apoptotic genes exclusively through the up-regulation of *Bax* and down-regulation of *BCL-2*.

### 2.6. Analysis of Radical Scavenging Effect

**Figure 12:**
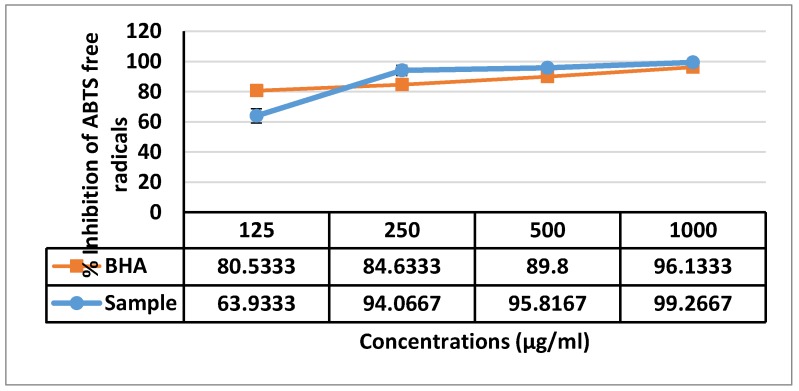
Inhibition activity of F13b1/PV-EA and comparison of a BHA group with treated samples. Data are expressed as mean ± standard division.

**Figure 13:**
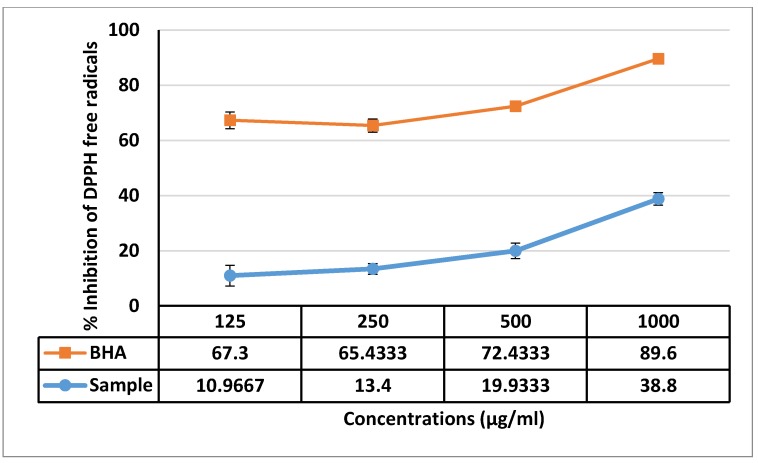
Shows F13b1/PV-EA radical inhibition activity and comparison of BHA group with treated samples. Data are expressed as mean ± standard division.

Examination to evaluate the antioxidant activity of F13b1/PV-EA, ABTS and DPPH free radical scavenging activity was performed. Figure 12 shows that F13b1/PV-EA demonstrated antiradical activity by inhibiting ABTS radical with IC_50_ values less than 125 µg/mL. F13b1/PV-EA displayed a dose-dependent activity and the ABTS scavenging effect has measured at 63% at a concentration of 125 µg/mL. In addition, pure compound displayed a dose-dependent activity and the DPPH scavenging effect was 38.8% at a concentration of 1000 µM. F13b1/PV-EA thus displayed a moderate inhibitory effect on DPPH free radicals (Figure 13).

### 2.7. Animal Study

#### 2.7.1. LD_50_ Tests (Lethal Dose 50 Test)

LD_50_ is the amount of a material that results in loss of life of 50% (one half) of animals in an experiment. The LD_50_ is one way to measure the short-term poisoning potential (acute toxicity) of a material. In this study three concentrations of F13b1/PV-EA were tested (12.5, 25 and 50 µg/mL) and in the concentration of 50 µg/mL, 50% of the mice died. This result indicated that a concentration of 50 µg/mL or higher was poisonous to mice and it was best for our main animal experiments to use concentrations of less than 50 µg/mL, which meant only using 12.5 and 25 µg/mL.

#### 2.7.2. Average Tumor Volume

To investigate the effects of F13b1/PV-EA in the inducement of apoptosis in Tubo breast cancer cells, mice were treated with two different concentrations of the compound (12.5 and 25 µg/mL), tamoxifen was used as a standard drug and one group without any treatment. At the end of the experiment, the mice were euthanized, tumors were excised from the mice and weighted. The tumor volumes (Table 1) were measured according to the formula below:

tumor volume = A × B^2^ × 0.5

where A: length, B: width.

Statistical analysis showed that the total tumor volume in all treatment groups was smaller than that of the control group.

#### 2.7.3. Histological Analysis

**Figure 14:**
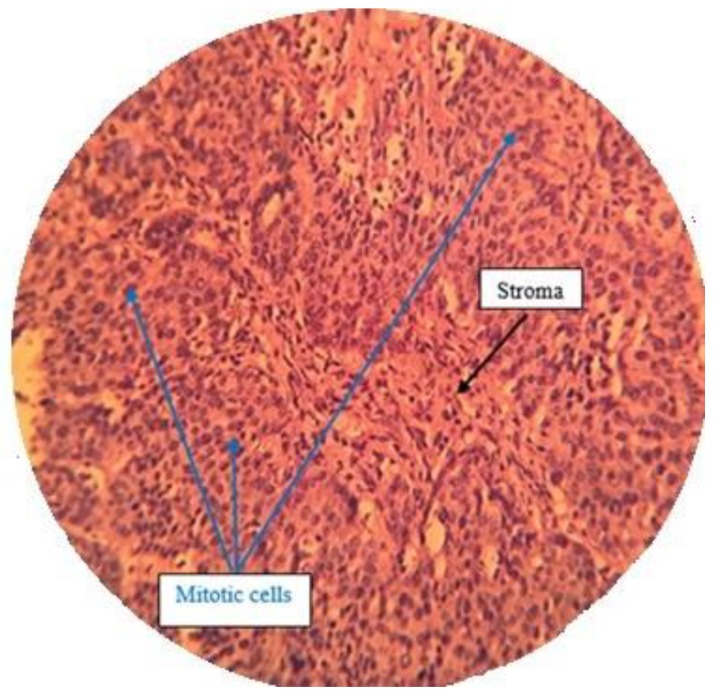
Negative control group with a high density of mitotic cells with dense nuclei (100×).

**Figure 15:**
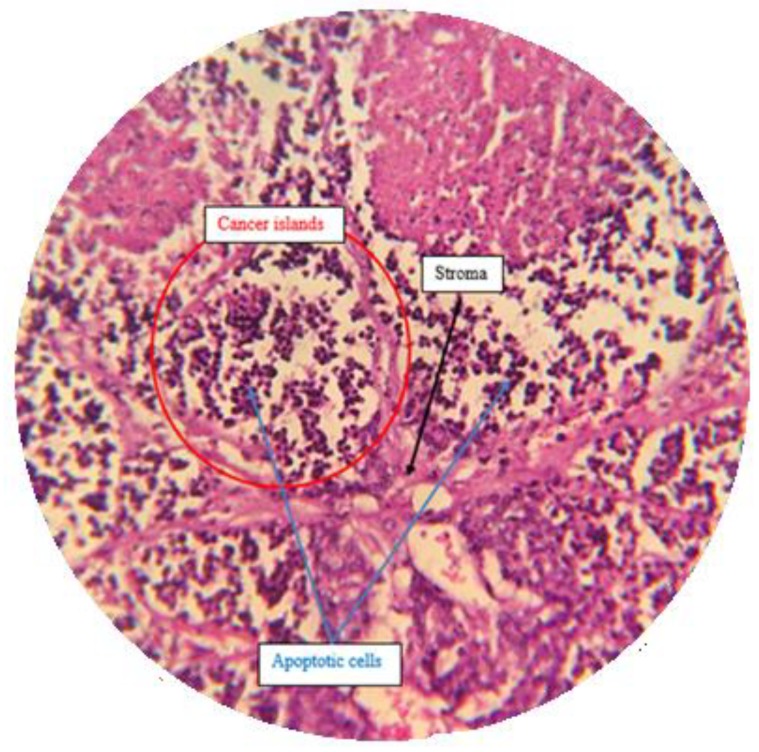
Standard drug control group with a high density of apoptotic cells with disintegrated nuclei in the cancer islands (100×).

**Figure 16:**
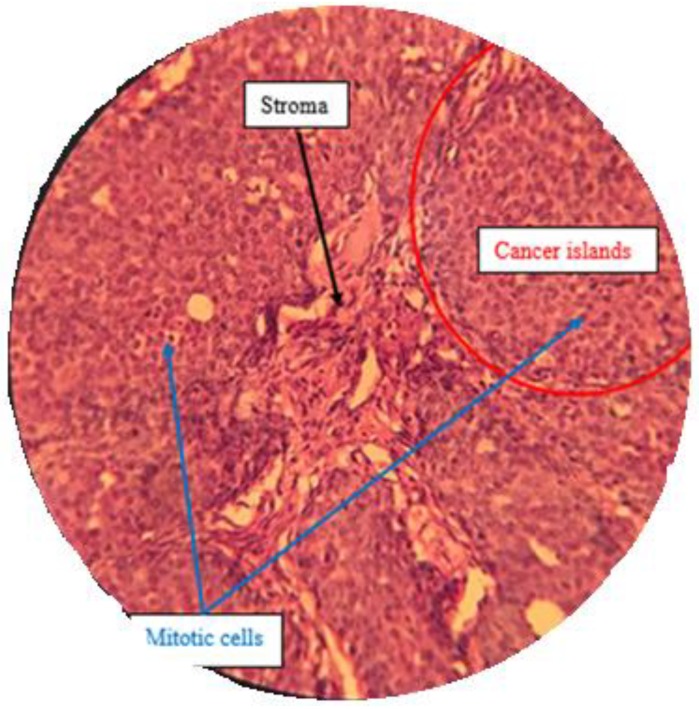
Low dose treatment group with a high density of mitotic cells with dense nuclei (100×).

**Figure 17:**
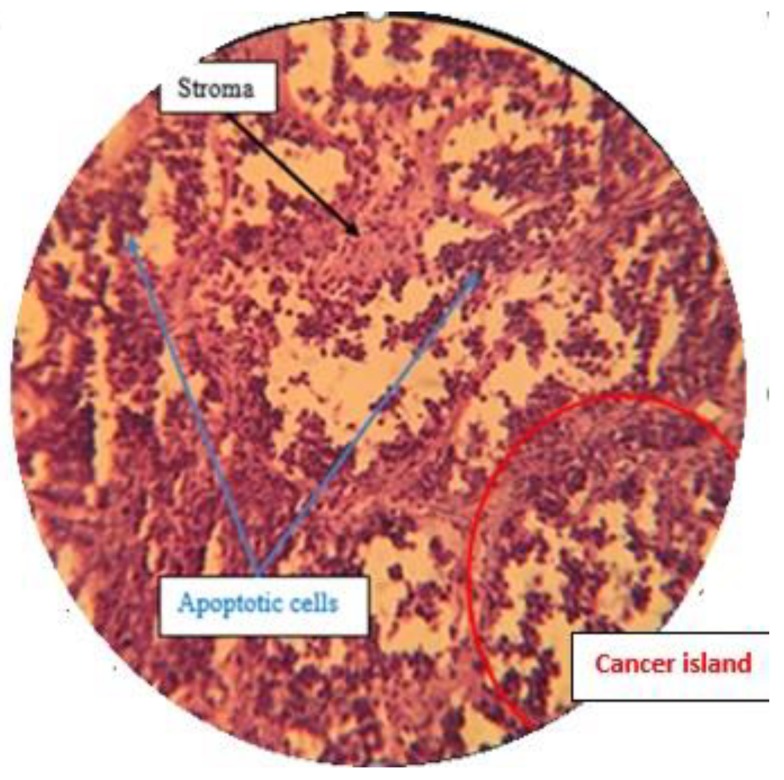
High dose treatment group with a high density of apoptotic cells with disintegrated nuclei in the cancer islands (100×).

A histopathological examination by H & E staining was done to confirm the effect of treatment by isolated compounds present in *Pistacia vera* hull extract F13b1/PV-EA. Five different sections from each H & E slide were monitored at 100× magnification (Figure 14, Figure 15, Figure 16 and Figure 17) and mean score was calculated from these five sections (Table 2). The scoring method is described in Table 4 in the Materials and Methods section.

## 3. Discussion

One of the unique characteristics of cancer is the capability of malignant cells to elude apoptosis [11,12]. Therefore, an all-inclusive perception of the apoptotic signaling pathways that are involved is of crucial importance for the discovery and development of target selective therapeutics. Mouse models are useful tool for carcinogenic study. They will greatly enrich the understanding of pathogenesis and molecular mechanisms for cancer. [13,14]. According to our previous study [10], the molecular and cellular response exerted by the PV-EA on the MCF-7 is noteworthy, thus it is vital to ascertain and determine the bioactive constituents that are present. Thus, the Isolation and identification of the bioactive compounds present were performed accordingly to help identify which compound(s) play a role in the safe and effective use for therapeutic purposes.

In this study, we managed to purify 14 fractions from the ethyl acetate extract of this plant in three steps and they were characterized using diverse spectroscopic analyses with subsequent confirmation using the HPTLC method. Chemical profiling of F13b1/PV-EA showed the presence of gallic acid and quercetin.

Phytochemical investigations conducted previously on the hulls of ripe pistachio have led to the identification of structurally varied secondary metabolites [15]. Barreca, et al. [9] in their research identified 20 derivatives from extracts of hull of pistachio, the most plentiful being gallic acid, followed by 4-hydroxybenzoic acid, protocatechuic acid, naringin, eriodictyol-7-*O*-glucoside, isorhamnetin-7-*O*-glucoside, quercetin-3-*O*-rutinoside, isorhamnetin-3-*O*-glucoside and catechin. The key difference between the red and green hulls was the presence of anthocyanins in the green hulls. For the first time, differently galloylated hydrolysable tannins, anthocyanins, and minor anacardic acids were identified. Thus pistachio hulls have structurally varied and potentially bioactive phenolic compounds [9].

One of the phenolic compounds is gallic acid (GA), chemically known as 3,4,5-trihydroxybenzoic acid [16]. Gallic acid is structured in such a way that it has phenolic groups that are a source of activated hydrogen atoms so that generated radicals can be delocalized over the phenolic moieties [17]. Another polyphenolic flavonoid compound is quercetin (3,3′,4′,5,7-pentahydroxyflavone) that is ubiquitous in plants and foods of plant origin. The most notable property of quercetin is its ability to act as an antioxidant. Quercetin seems to be a strong flavonoid for defending the body against reactive oxygen species, which is very important in cancer therapy [15].

Young et al. [18] have examined polyphenols as potential inhibitors of UGDP-glucose dehydrogenase (UGDH) activity. Gallic acid and quercetin decreased the specific activity of UGDH and inhibited the proliferation of MCF-7 human breast cancer cells. Western blot analysis showed that gallic acid and quercetin did not affect UGDH protein expression, suggesting that UGDH activity is inhibited by polyphenols at the post-translational level. Kinetics studies using human UGDH revealed that gallic acid was a non-competitive inhibitor with respect to UDP-glucose and NAD^+^. In contrast, quercetin showed a competitive inhibition and a mixed-type inhibition with respect to UDP-glucoseand NAD^+^, respectively. These results indicate that gallic acid and quercetin are effective inhibitors of UGDH that exert strong antiproliferative activity in breast cancer cells.

By evaluating the cytotoxic properties of F13b1/PV-EA on MCF-7 cell line, it was observed that there was decreased cell viability in tandem with increasing concentration and time of treatment. Multiple papers about gallic acid and its pharmacological activities have been published. Gallic acid has shown some activities that include in the following: angiogenesis, repression of cell viability and reproduction in human glioma cells, prevention of the propagation of HeLa cervical cancer cells, inhibition of ribonucleotide reductase, induction of apoptosis in humoral cell lines, prevention of lymphocyte duplication and cyclooxygenases in human HL-60 promyelocytic leukemia cells, stimulation inactivating phosphorylation via ATM-Chk2 activation and anti-oxidant activity [11,18,19].

The effects of three different doses of F13b1/PV-EA on MCF-7 was evaluated through acridine orange/propidium iodide (AO/PI) staining and fluorescence microscopy. The tests confirmed that the compound has a dose-dependent effect on cell viability and induces apoptotic morphological changes in the treated cells. The results show reduced viability with the presence of higher levels of apoptotic cells for all three treatment concentrations.

To get a better understanding of the efficacy of the bioactive compound on the nucleus, treated cells were stained with Hoechst stain. The cells was seen to have gone through major nuclear changes upon treatment. In the control, the cells were uniformly stained via the fluorescent Hoechst stain, indicating the nuclei of the cells were intact. However, with increasing concentrations of the compound, there was a growth of severity seen captured on the fluorescence signals and luminous points where the cells exhibited apoptotic morphological changes.

By utilizing PI staining, it was established whether the MCF-7 cells treated with F13b1/PV-EA underwent apoptosis and if it was accompanied by notable alterations in the cell cycle, and the distribution index was also investigated via PI staining. This was accompanied by growth in the Sub-G1 population with increasing concentrations. This cells population possessed a sub-diploid DNA content which is indicative of DNA fragmentation occurring during apoptosis. F13b1/PV-EA was thus shown to be able to bring about apoptosis by changing the regulation of apoptotic genes, particularly through up-regulation of *Bax* and down-regulation of *BCL-2*.

*Caspase 3*, *Caspase 8*, *CAT*, *Bax* and *SOD* genes expression increased compared to control (gene expression in cancer cells without any treatment). Further investigations revealed that compound treatment eventually decreased the expression level of Bcl2.

The cytotoxicity and anti-cancer effects of hydro-alcoholic extracts of pistachio shell on HepG2 and L929 cells was elucidated by Harandi et al. [20]. Cell viability of HepG2 and L929 was decreased after 24 and 48 h of treatment with IC_50_ 1500 and 1000 μg/mL for HepG2 and 2000 and 1500 μg/mL for L929. *Bax* and *P53* genes were shown to be up-regulated and *Bcl-2* gene was displayed to be down-regulated after treatment.

ROS are stabilized by reactions which in turn cause cellular damage and the formation of carcinogenic DNA adducts. The consumption of antioxidants has been shown to reduce the risks of getting cancer. Our compound displayed a dose dependent activity on ABTS and a slow inhibitory effect on DPPH free radicals.

Hashemi, et al. [21] investigated the antioxidant activity of a *Pistacia atlantica* extract. The antioxidant activity of the extract was 4.6 ± 0.66 μg/mL, while it was 25.41 ± 1.89 μg/mL for butylated hydroxytoluene (BHT). The total phenol, flavonoid and flavanol contents were 269 mg GAE/g, 40.7 mg RUT/g and 88.12 mg RUT/g, respectively. In recent times, identifying and an emphasis on chemical agents and natural products with the capability of preventing human cancer has been an important objective in preclinical cell culture and animal efficacy testing models. In clinical chemoprevention tests, according to the toxicity screening experiments, only the most active factors have potential as human chemopreventives.

Our animal experiment study results showed that the total tumor volume of all treatment groups were smaller than that those in the control group. According to several papers, quercetin has anti-proliferative effects to cancer, enhances the efficacy of chemotherapeutic factors, in vivo lymphocyte tyrosine kinase prevention and anti-tumor activity. LD_50_ tests in rats showed that injection of boldine remarkable decreased breast cancer tumor size and at dose of 100 mg/kg body weight was well tolerated.

*Ferulago angulata* leaf hexane extract (FALHE) is capable of inducing apoptosis on MCF-7 cells. An in vivo study showed that FALHE reduce the tumor size from 2031 ± 281 mm^3^ to 432 ± 201 mm^3^ after treatment. Acute toxicity tests revealed an absence of toxic effects of the two compounds on mice [22].

This study explained the potential use of red *Pistacia vera* hull as chemopreventive drug, as it can be exploited as a new lead compound for prodrug therapy. Since MCF7 is an estrogen-receptor negative human breast cancer, it’s good for potential future examination of our extract and its active compound in another estrogen receptor-responsive cell line like MDA-231 [23]. Also as extracts of plants may activate the immune system of the hosts and kill cancer cells, one can consider testing the concentrations of various cytokines, tumor necrosis factor, etc. [24], before and after administration of PV. On the other hand, since 100% pure gallic acid and quercetin are commercially available, a combination of these two agents at the same concentration ratio as they exist in pistachio can be tested to examine the anti-tumor effect of PV extract. These results present an opening to new roads for discovery of anticancer drugs and treatment of cancer by promoting induced apoptosis.

## 4. Materials and Methods

### 4.1. General Experimental Procedures

Column chromatography (CC) was run on silica gel 60 column (Merck, Darmstadt, Germany). Thin layer chromatography (TLC) was performed on an aluminum supported silica gel 60 (Merck). The compound purity confirmation was confirmed on a HPTLC system (Gilson, Inc., Middleton, WI, USA) with a mobile phase of methanol (100%). Gas chromatography was performed on a Breeze2 system.

### 4.2. Collection and Extraction of Plant

**Figure 18:**
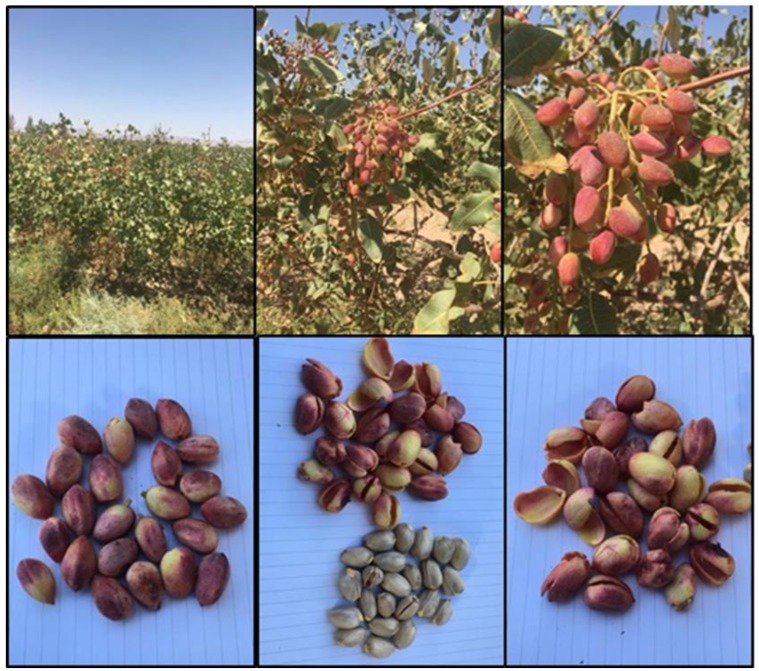
*Pistacia vera* tree, fruit and red hulls.

**Figure 19:**
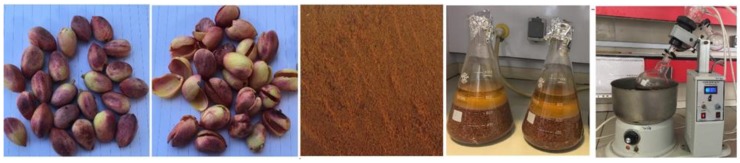
PV extraction method steps: deshelling, drying, maceration, evaporation and extraction.

The hulls of the *Pistacia vera* (PV) were procured from Kerman Province, Iran, and identified at the Herbarium in the Institute of Biological Science, University of Malaya, by Dr. Yong Kien Thai with voucher number KLU48697. The hull after drying and powdering was soaked in ethyl acetate. The extract was filtered from the residue by using filter paper and the residue was re-extracted with ethyl acetate solvent twice more. By using a rotary evaporator (R110 Rotavapor, Buchi Labortechnik AG, Flawil, Switzerland), the solvent was evaporated at a temperature of 40 °C, giving a dark brown crude extract and stored in 4 °C before further testing was done (Figure 18 and Figure 19).

### 4.3. Bioassay-Guided Fractionation of Pistacia vera Ethyl Acetate Extract

*Pistacia vera* ethyl acetate extract was chosen for next analysis and purification according to the data from our previous study. PV-EA (16 g) was subjected to column chromatography using a glass column (60 cm L × 6 cm I.D) packed with Merck Kieselgel 60 stationary phase. Briefly, the silica gel was made into a slurry with solvent before it was packed into the column and it was allowed to equilibrate for at least one hour before use. The extract was then introduced on top of the silica surface. The column was generally eluted with combinations of solvents with a stepwise increase in the solvent polarities. In the first step hexane and ethyl acetate (70:30) was used as solvent. Isolated fractions were monitored by TLC and those samples displaying similar Rf values on the TLC were pooled to yield 14 fractions (designated F1-F14). The MTT cell viability assay was carried out on these 14 fractions for choosing the most cytotoxic fraction, which was fraction number 13 (F13).

In the next step (step 2), fraction number 13 (F13) was subjected to glass column (60 cm L × 6 cm I.D.) chromatography with combinations of ethyl acetate and dichloromethane of increasing polarity. Isolated fractions were monitored by TLC and finally seven appropriate fractions (F13a-F13g) were combined and dried. After an MTT assay the most cytotoxic fraction was fraction number 2. In the last step of fractionation (step 3), fraction number 2 (F13b) was subjected to column chromatography with combinations of dichloromethane and methanol of increasing polarity. After TLC analysis on the isolated fractions, four appropriate fractions (F13b1-F13b4) were combined and dried for the next MTT assay. At the final stage, fraction number 1(F13b1) of about 10 mg was selected as the most effective fraction or pure compound.

### 4.4. Cell Lines and Cell Culture

The MCF-7 human breast adenocarcinoma cell line was procured via the American Type Culture Collection (ATCC, Manassas, VA, USA). Roswell Park Memorial Institute medium (RPMI-1640) supplemented with 10% fetal bovine serum and 1% penicillin and streptomycin (Sigma-Aldrich, St. Louis, MO, USA) was used for the cultivation of MCF-7 and this were subsequently cultured in a humidified incubator using 5% CO2 at 37 °C.

### 4.5. MTT Cell Proliferation Assay

Briefly, 24 h prior to treatment, MCF-7 cells (5 × 10^4^ cells/mL) were seeded in a 96-well plate. Dissolved compounds in RPMI were used in various concentrations (from 7.8 to 500 μg/mL). After 72 h in each well of plates was added 20 µL of MTT solution and then plates were incubated for further 4 h. In the next step, 150 µL of DMSO was put into each well and incubated for 10 min to solve the purple formazan crystals. The dose-response curves were mapped to obtain IC_50_ values and identify the best active fractions or pure compound.

### 4.6. High-Performance Thin Layer Chromatography (HPTLC) Analysis

High-performance thin-layer chromatography is an improved form of the normal thin-layer chromatography. Several augmentations can be made to the basic method of thin-layer chromatography to automate the different steps, increase the resolution achieved and allow more accurate quantitative measurements. In this method 10 tracks were applied on the TLC plate with silica gel 60 (for 10 × 10 cm) using micro syringe. The plates were saturated for 20 min in a twin trough glass chamber with the mobile phase of methanol (100%). The plates were placed in the mobile phase and dried. A densitometric scanning of plates were performed at 254 nm and 320 nm using a Camag TLC scanner III operated in reflectance–absorbance mode. To examine the chemical profiling of F13b1/PV-EA, analysis was carried out using a C_18_ column on a Breeze2 system. First, 20 μL of blended standard (gallic acid, cyaniding and flavonoid quercetin) was injected to the column with water and acetonitrile solvent. Subsequently, 20 μL of standard of gallic acid and finally 20 μL of compound or F13b1/PV-EA was injected to the column. For comparing the three injections’ properties and identification of the chemical profile of our compound, after each injection, the retention time (RT) or the amount of time that a compound spends on the column from injection to detection, was calculated.

### 4.7. Flow Cytometry Analysis

In the next step, three different concentrations (8, 16 and 32 µg/mL) of F13b1/PV-EA was added for 2 days to MCF-7 cells that were seeded (5 × 10^5^ cells/well) in a 35 mm dish for 24 h. Then nuclear fractions from the cells were obtained according to the kit’s propidium iodide staining protocol. The intensity of the fluorescence was detected using a FAC Scan flow cytometer (BD Biosciences, San Jose, CA, USA) and analyzed via Cell Quest software.

### 4.8. Acridine Orange/Propidium Iodide Staining (AO/PI)

After a 24-h incubation of the 1 × 10^6^ cells/well of MCF-7 cells in a 6-well plate, the cells were treated with 8, 16 and 32 µg/mL of F13b1/PV-EA for 48 h. Then detached cells were dyed with AO/PI stain according to manufacturer’s protocol and examined using fluorescence microscope.

### 4.9. Hoechst 33342 Staining

Using a functional vital dye the classical morphological criteria, the quantification and determination of cell death notation was carried out. The MCF-7 cells were treated using three different concentrations (8, 16 and 32 µg/mL) of F13b1/PV-EA for 48 h. Hoechst 33342, which is a specific stain used for AT-rich regions of double-stranded DNA was utilized. The cells were incubated for 15 min with Hoechst 33342 dye (5 μg/mL in PBS)) and subsequently visualized using a BHZ, RFCA microscope (Olympus, Tokyo, Japan) equipped with a fluorescent light source with an excitation wavelength of 330 nm and a barrier filter of 420 nm.

### 4.10. Gene Expression Assay

By employing the RT-PCR, the gene expression of *Bax*, *Bcl2*, *Caspase 3*, *Caspase 8*, *CAT* and *SOD* were analyzed. RNA was extracted from MCF-7 cells (3 × 10^6^ cells/well) that were treated with 15 µg/mL of F13b1/PV-EA for 24 h, using the manufacturer’s instructions for RNA extraction. The mRNA was transcribed in reverse to cDNA by adhering to the manufacturer’s protocol using the Advantage RT-PCR kit. cDNA was amplified via a real time and sybr green kit. Table 3 shows the specific primers used for amplifying the cDNA.

### 4.11. DPPH Radical Scavenging Assay

The free radical scavenging activity of F13b1/PV-EA was assessed based on its effect trapping 2,2-diphenyl-1-picrylhydrazyl (DPPH) free radicals. 0.1 mM methanolic DPPH solution was mixed with the varying concentrations of F13b1/PV-EA (125, 250, 500 and 1000 µg/mL), in an equal volume. After 30 min of incubation, the absorbance of the samples was read at 517 nm. In the control group water and BHA was used as a standard compound. The percentage of inhabitation of DPPH free radical was calculated according to the formula below:

Percentage of inhibition of DPPH free radical = Absorbance of control − Absorbance of sample/Absorbance of control × 100

### 4.12. ABTS Radical Scavenging Assay

In brief 1 mL of various concentrations of F13b1/PV-EA (125, 250, 500 and 1000 µg/mL), were mixed with 1 mL of ABTS·+ working solution. After incubation period of 1 h in room temperature in the dark, absorbance was read at 734 nm. To prepare the ABTS·+ stock solution, 7 mM of ABTS and 2.45 mM of potassium persulfate were mixed, incubated at room temperature for 12–16 h and finally ABTS·+ stock solution was diluted with distilled water to gain 0.70 ± 0.02. Percentage of inhibition of ABTS free radical was calculated according to formula in previous part.

### 4.13. Experimental Animals

The experiments on the animal were divided into 2 parts. First, the lethal dose 50% (LD_50_) test and after that in vivo anti-tumor assessment was carried out. For LD_50_ testing and to determine the acute toxicity of the proposed compounds, 18 healthy male Balb/C mice (25 ± 5 g, five-weeks-old) were provided by the animal house of the University of Malaya Animal Experimental Unit (AEU), in clean, sterile and polyvinyl cages. The mice were maintained under standard conditions, temperature of 22–26 °C, 45–50% relative humidity with water, food and sterile diet under pathogen-free environment and maintained on a 12 h light/dark photo period. For the second part or in vivo anti-tumor assessment, 24 healthy female Balb/C mice were purchased from Pasteur Institute of Mashhad (Iran). The mice were maintained under conditions that were mentioned above. The animal studies were performed after approval of the protocol by the FOM Institutional Animal Care and Use Committee, University of Malaya (FOM, IACUC, ethic No.: 2016-190405/IBS/R/MS).

#### 4.13.1. Lethal Dose 50% (LD_50_) Test

LD_50_ is the amount of the substance (usually per body weight) required to kill 50% of the test population within a specific time. In order to observe the overall effect of our compound on a living subject, 18 male Balb/C mice were divided into three groups of animals in each group. Every group was treated with one concentration of F13b1/PV-EA, 1 time every week until 2 weeks, for a total of two times. Three doses (12.5, 25 and 50 µg/mL) of pure compound was dissolved in 10% tween 20 and given orally by gavage to the mice. For purpose of calculating the consumable dose (dosage compound for each mouse) we used the formula below:

(Concentration of the compound) × (mouse body weight) × 6 (the rate of metabolism mice to the human) = consumable dose

Animals were monitored for 30 min, 2, 4, 8, 24 and 48 h for up to two weeks after the dosing. Any signs to the animals involving toxicity and/or mortality and behavior changes were observed keenly and recorded throughout the experimental period for 14 days.

#### 4.13.2. In Vivo Anti-Tumor Assessment

To construct an allograft breast carcinoma model, Tubo cancer cell lines were grown and harvested under appropriate conditions. Tubo cells are a cloned cell line established in vitro from a BALB-neuT mouse mammary carcinoma. 1.5 × 10^6^ cells were suspended in 0.2 mL PBS and were injected this subcutaneously into the right flank of BALB/c mice (*n* = 6). After the tumor inoculation (approximately 7 days later) the animals were randomly divided into four groups of six mice:

Group 1: Negative control (just received 10% tween 20 orally) once daily for 2 weeks.

Group 2: Standard drug control group as a positive control (Tubo induced + tamoxifen 10 mg/kg dissolved in 10% tween 20 via oral administration) once daily for 2 weeks.

Group 3: Experimental group A (Tubo induced + low dose of compound dissolved in 10% Tween 20 via oral administration) once daily for 2 weeks.

Group 4: Experimental group B (Tubo induced + high dose of compound dissolved in 10% Tween 20 via oral administration) once daily for 2 weeks.

**Figure 20:**
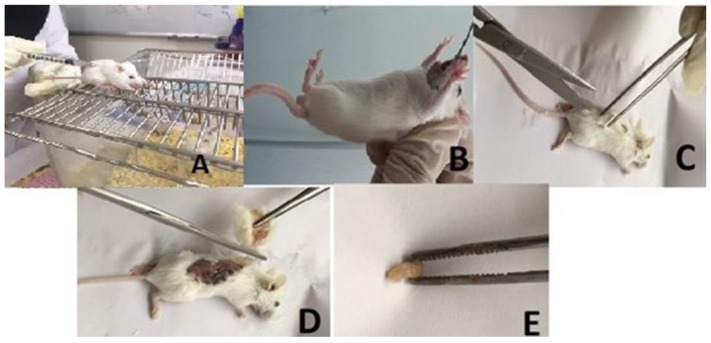
In vivo anti-tumor assessment in mouse. (**A**) breast cancer injection; (**B**) oral gavage treatment; (**C**,**D**) tumor isolation and (**E**) tumor mass.

For calculating the consumable dose of F13b1/PV-EA in the experimental groups, we used two concentrations (12.5 and 25 µg/mL) based on the LD_50_ result. The mice were euthanized at the end of 14 days treatment period and the tumors were then excised and weighted (Figure 20). The tumor volumes were measured according to the formula below:

tumor volumes = A × B^2^ × 0.5

where A: length, B: width

H & E staining was performed in order to analyze the histopathological samples.

#### 4.13.3. Histological Analysis and Scoring Method

After separating tumor from the body, it was instantly was fixed in 10% formalin overnight, embedded in paraffin, cut into 4 µm sections and stained with hematoxylin-eosin (H & E). Tissue scoring was conducted according to Elston and Ellis method [25], with some slight modifications. Randomly five different sections from each H & E slide view at 100× magnification and mean score is calculated from these five sections and the scoring is referred to in Table 4. Only structures which could not be misinterpreted as anything except mitotic figures were taken into account. For apoptosis only hyperchromatin or pyknotic nuclei with hollow signs were counted:

Mitotic index = The numbers of mitotic cells/the number of total cells

Apoptotic index = The numbers of apoptotic cells/the number of total cells

### 4.14. Statistical Analysis

For statistical assessment *t*-test was applied for non-dose responses. Dose response experiments were studied using the Omnibus test followed by Rodger’s method. All values are declared as mean ± S.D. Probability values * *p* < 0.05 was considered as statistically significant.

## 5. Conclusions

To conclude, the results from the present study provide an understanding of the cytotoxic effect of *Pistacia vera* red hull. Cytotoxicity studies of *Pistacia vera* red hull ethyl acetate (PV-EA) extract on different cancer cell lines and subsequent chemical purification of bioactive PV-EA have led to the isolation of gallic acid and quercetin. Purified compound (F13b1/PV-EA) was shown to possess cytotoxic effects against MCF-7 cells. This finding may have an impact on the future of cancer treatment by providing another positive avenue for the discovery of a combined anticancer drug treatment that may promote induced apoptosis.

## Figures and Tables

**Table 1:** The tumor volume of Tubo cancer cells in the four treatment groups.

Group	Tumor Volumes
Negative control group	10.7 ± 1.2 cm^3^
Positive control group	0.95 ± 0.7 cm^3^
Experimental group A(12.5 µg/mL)	2.5 ± 0.8 cm^3^
Experimental group B (25 µg/mL)	0.8 ± 0.7 cm^3^

**Table 2:** Apoptotic index, and mitotic index in animal treated with F13b1/PV-EA.

Experimental Group	Apoptotic Index	Mitotic Index
Negative control	1 (2 ± 1)	1 (15 ± 3)
Positive control	2 (9 ± 0.5)	1 (4 ± 0.8)
Low-dose	2 (5 ± 0.1)	1 (9 ± 1)
High-dose	3 (11 ± 0.9)	1 (5 ± 0.7)

**Table 3:** Primer sequence for amplifying cDNA.

*BCL-2*	5 CATGTGTGTGGAGAGCGTCAA 3 F 5 CAGATAGGCACCCAGGGTGA 3 R
*BAX*	5 TTTGCTTCAGGGTTTCATCCA 3 F 5 CTCCATGTTACTGTCCAGTTCGT 3 R
*Caspase 3*	5 GTGGAACTGACGATGATATGGC 3 R 5 CGCAAAGTGACTGGATGAACC 3 R
*Caspase 8*	5 CTGGGAAGGATCGACGACGAT 3 F 5 CATGTCCTGCATTTTGATGG 3 R
*CAT*	5 CTTCCCGCTTGAATGTGAAG 3 F 5 CCGATTACATAAACCCATCA 3 R
*SOD*	5 GCTCCTAAGCCGCTTACGGTT 3F 5 CACGCCATCGGCATTGGCAAT 3R

**Table 4:** Scoring method for apoptosis and mitotic indexes.

Apoptotic Index		Mitotic Index	
Apoptotic Count	Score	Mitotic Count	Score
0–4	1	<25	1
5–10	2	25–50	2
11–15	3	51–100	3
>15	4	>100	4

Adapted from Elston and Ellis [25], with some slight modifications.
